# PTGR1 is involved in cell proliferation in thoracic ossification of the ligamentum flavum

**DOI:** 10.1371/journal.pone.0292821

**Published:** 2023-11-01

**Authors:** Kuankuan Liu, Li Shu, Ann Yehong Huang, Yanan Chang, Zhongqiang Chen, Chi Zhang

**Affiliations:** 1 Central Laboratory, Peking University International Hospital, Beijing, China; 2 Department of Biochemistry, University of Texas Southwestern Medical Center, Dallas, Texas, United States of America; 3 Department of Orthopedics, Peking University International Hospital, Beijing, China; 4 Biomedical Engineering Department, Peking University, Beijing, China; Augusta University, UNITED STATES

## Abstract

Thoracic ossification of the ligamentum flavum (TOLF) is a heterotopic ossification of spinal ligaments, leading to serious myelopathy. TOLF underlying mechanisms are not well understood. Our iTRAQ analysis have identified ten inflammatory factors related to TOLF, including l. We found that PTGR1 expressions increased in TOLF by RT-PCR and western blot in this study. Both cell proliferation and differentiation are important for the process of bone formation. In our previous study, we demonstrated that TOLF primary cells grew faster than control cells. It was reported that knockdown of PTGR1 inhibited cell proliferation. We hypothesize that PTGR1 may participate in cell proliferation in TOLF. To test this hypothesis, TOLF primary cells were treated for 24h with PTGR1. We observed that PTGR1 increased cell proliferation. The effect of PTGR1 on cell proliferation related genes was examined in TOLF primary cells. Our results showed that PTGR1 was able to activate expressions of c-Myc and CyclinD1. Moreover, blocking JNK pathway by selective JNK inhibitor SP600125 eliminated the positive effect of PTGR1 on c-Myc expression, indicating that PTGR1 activated the expression of c-Myc via JNK pathway. Our new findings suggest that PTGR1 is involved in cell proliferation of TOLF.

## Introduction

The ligamentum flavum is the primary structure to keep the spine stability, extending & flexing, and protecting the spinal cord [1]. Thoracic ossification of the ligamentum flavum (TOLF) is a heterotopic ossification of spinal ligaments. TOLF progresses over a long period of time, resulting in devastating spinal cord injury that leads to serious myelopathy [2–4]. It has been reported that conservative treatments are ineffective in most cases [5]. Surgical decompression has to be used to treat TOLF eventually with potential complications.

The incidence of TOLF increases with age, consistent with the characteristics of chronic and degenerative diseases [6]. Most cases of thoracic spinal stenosis are caused by the ossification of the ligamentum flavum. With a prevalence rate between 3.8 and 63.9%, it is prevalent primarily in East Asian countries, such as China and Japan [3, 7, 8]. However, the pathogenesis of TOLF has not been well elucidated, which limits its pharmacological treatment. There is a necessity for research to identify potential factors involved in TOLF to explore possible underlying molecular mechanisms.

The etiology of TOLF has been addressed by different studies. Mechanical effects, inflammatory factors, and genetic factors have been gradually considered as potential factors associated with TOLF [9–11]. In accordance with the magnetic resonance imaging lesion distribution, the most common site of TOLF is the lower thoracic spine T10-T12 [6]. Compared with single-level lesions, multi-level TOLF presents with a wide ossification of both immobile and mobile segments that differs in progression and clinical presentation from single-level lesions [12–14]. TOLF progression might be partially affected by local abnormal mechanical stress [4]. Previous reports showed that the involvement and possible mechanism of tumor necrosis factor α (TNF-α) in TOLF, suggesting the contribution of TNF-a in TOLF [10]. IL-6 has recently been identified to be involved in TOLF [11]. It was reported that inflammatory cytokine IL-6 increased in the culture supernatant of TOLF primary cells. IL-6 was able to activate expressions of osteoblastic factors including BMP2 and OSX. Expression of cell proliferation factor cyclin D1 also increased in the presence of IL-6. IL-6 mediated the BMP2 activation through p38 MAPK pathway. These recent data provide evidences that inflammatory factors participate in TOLF.

For the purpose of obtaining a comprehensive and quantitative protein profile, a new approach, called isobaric tags for relative and absolute quantitation (iTRAQ), has been developed for investigating proteomic changes throughout development. In our previous study, TOLF patients’ ligamentum flavum protein profile was examined using iTRAQ-based quantitative proteomics [10]. The fold change cutoff ratios were ≥1.5 for up-regulation and ≤0.67 for down-regulation. Total 282 proteins showed differential expression by iTRAQ. Ten inflammation-related proteins have been identified among these proteins, including Tumor necrosis factor (TNF-a), Insulin-like growth factor II, Insulin-like growth factor-binding protein 5, Prostaglandin reductase 1 (PTGR1), Latent-transforming growth factor beta-binding protein 3, Transforming growth factor beta-1, Neutrophil elastase, Serum amyloid A-4 protein, Protein S100-A9, and Prostaglandin-H2 D-isomeras [10]. It is known that PTGR1 is a NAD(P)H-dependent oxidoreductase that inhibits pro- and anti-inflammatory eicosanoids through metabolic inactivation. Recent researches indicated that PTGR1 may play a role in hepatocellular carcinoma, prostate cancer and non-small cell lung cancer [7, 8, 15]. It suggested that PTGR1 could be a potential therapeutic target for cancer [16]. Yet to date, the role of PTGR1 in TOLF is not clear.

In this study, we aimed to explore the potential effect of PTGR1 on TOLF and elucidate the possible molecular mechanism.

## Materials and methods

### Patients and ligament samples

Patients were recruited from Department of Orthopedics in Peking University Third Hospital between August 1, 2015 and May 31, 2017 as previously described [17]. To get proteomic data for TOLF patients, normal ligamentum flavum in adjacent levels was used as the control. During the operation, the ossified ligamentum flavum of TOLFs (OS-Lig) and control ligaments (Lig) were carefully collected from patients. In this study, proteins were extracted from ossified versus normal ligamentum flavum.

### Cell culture

The primary cells of TOLF (OS-Lig) and ligamentum flavum control (Lig) were obtained from TOLF patients. The ligamentum flavum in the control site and the ossified site were aseptically dissected during surgery through the use of a dissecting microscope. The surrounding tissues were carefully removed as previously described [18]. The ligaments were rinsed with PBS after dissection. In the following digestion, 0.25% trypsin (Gibco, Grand Island, NY, USA) was used at 37°C for 1h, followed by 200 U/ml type I collagenase (Sigma-Aldrich, St. Louis, MO, USA) at 37°C for 4h. Cells were cultured in DMEM (Hyclone, Logan, UT, USA) supplemented with 10% fetal bovine serum, 100 U/mL penicillin, and 100 mg/mL streptomycin (Gibco) at 37°C in a humidified atmosphere containing 5% CO_2_.

### Cell proliferation assay

The primary ligamentum flavum cells were seeded in 96-well culture plates at initial density of 1×10^4^ cells/well overnight. Cells were treated with various concentrations of PTGR1 for 24h as indicated. Cell proliferation was examined by the Cell Counting Kit-8 (CCK8) kit (Dojindo, Japan) following the manufacturer’s instructions. The cells were stained with 10μl CCK8 dye in 90μl culture medium at 37°C for 2 h. Microplate reader was used to determine the absorbance at 450 nm.

### Protein extraction and iTRAQ

Using the Bradford method after protein extraction, the protein concentration was determined following the manufacturer’s protocol (Bio-Rad laboratories, Hercules, California, USA). Each sample was mixed with 200ug of Reducing Reagent and 2ul of Cysteine-Blocking Reagent, then added 100 μl Dissolution Buffer to iTRAQ kit as previously described [17]. Bottom solution was discarded. The process was repeated three times before 4 μg of trypsin was dissolved in the acquired solution using a new collection tube. As a result of combining the 50 μl Dissolution Buffer 5 with the above solutions, the digested sample reached 100μl. Labeling of the samples was performed by adding iTRAQ reagents from the Reagent-8Plex Multiplex Kit to the samples, and then labeling with iTRAQ according to the manufacturer’s instructions (Applied Biosystem). Accordingly, we considered proteins with higher iTRAQ ratios>1.5 to be up-regulated, while proteins with lower iTRAQ ratios<0.67 were down-regulated.

### Western blot

Protein expression was detected using western blot analysis as previously described [19]. Proteins were separated by 12.5% SDS-PAGE and transferred onto PVDF membranes (Millipore) by electroblotting. The blot was blocked in 5% skim milk with TBST for 1h and then incubated overnight at 4°C with primary antibody at a 1:1000 dilution. The blots were washed three times in 1 × TBST for 10 minutes. The blot was incubated with secondary antibody for 2h at room temperature. Protein bands were visualized by enhancing chemifluorescence detection system. Primary antibody included as below: c-Myc (1:1000, Abcam), PTGR1 (1:1000, imm) and B-actin (1:1000, Abcam).

### Real-time quantitative PCR

Total RNA was extracted from cells by column-based extraction (RNeasy Mini kit, Qiagen). RNA purity and integrity were determined by the RNA 6000 Nano assay with an Agilent Bioanalyzer 2100 (Agilent Technologies, Santa Clara, CA) as previously described [20]. RNA was reverse transcribed into cDNA using primeScript RT Master Mix (TAKARA). The qPCR was performed using TB Green II and the reaction system was as follows: 10 μL of mix, 2μL each of forward and reverse primers, 3 μL of cDNA, and 5 μL of TB. Specific primers for PTGR1, Cyclin D1 and c-Myc were designed using Primer Premier (Premier Biosoft, Palo Alto, CA, USA) and ordered by Sangon Biotech (Shanghai, China). The reaction conditions were as follows: 95°C for 30 sec, 40 cycles of 95°C for 10 sec and 60°C for 30 sec. Data were reported as cycle threshold (Ct) values. The RNA levels were compared using the 2- ^ΔΔ^Ct method and were normalized to GAPDH levels.

### Statistical analysis

All experiments were performed in triplicates. Statistical analysis was performed with GraphPad Prism version 8.0. 1, and quantitative data are presented as the mean ± standard deviation. Student’s t test was used to compare groups, and p≤0.05 was considered significant.

## Results

### The level of PTGR1 expression increased in TOLF

**Fig 1:**
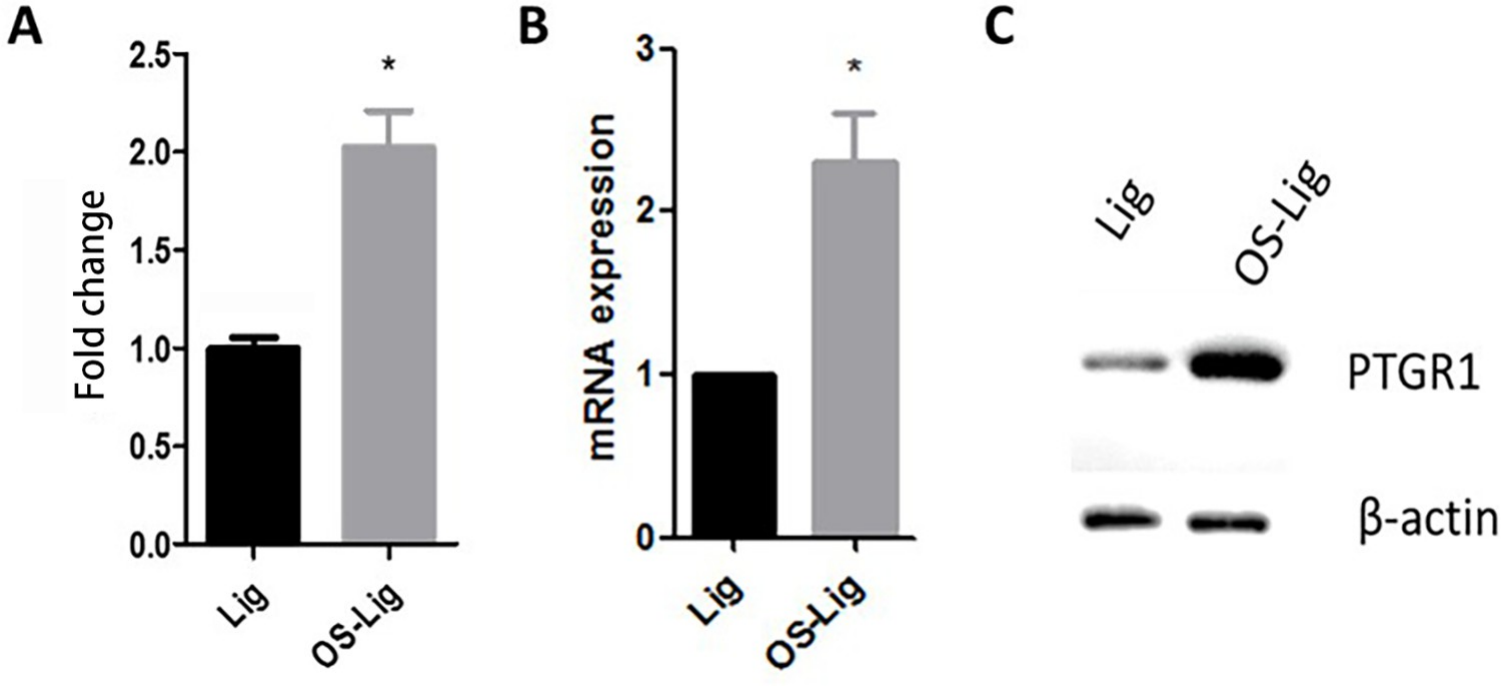
The expression levels of PTGR1 in OS-Lig were higher than Lig. A) Fold change of PTGR1 protein in the ossified ligamentum flavum of TOLF. Proteins were extracted from the ossified ligamentum flavum (OS-Lig) and normal ligamentum flavum (Lig). The samples were then labeled with iTRAQ following the manufacture’s protocol. * p <0.05 compared with the Lig group; B) Relative PTGR1 mRNA expression was detected using qPCR in primary cells, primary cells were derived from the ossified ligamentum flavum (OS-Lig) and normal ligamentum flavum (Lig); C) PTGR1 protein increased in primary cells of the ossified ligamentum flavum of TOLF by western blot. Primary cells were derived from the ossified ligamentum flavum (OS-Lig) and normal ligamentum flavum (Lig). Protein was purified and detected by western blot. An anti-PTGR1 rabbit monoclonal antibody (1:1000) or an anti-β-actin rabbit monoclonal antibody (1:200) was used.

Proteins of OS-Lig tissues and Lig tissues from patients with TOLF were quantified by iTRAQ analysis. We found ten inflammatory proteins of interest. Among these proteins, the PTGR1 expression levels of OS-Lig tissues were twice higher than Lig tissues by iTRAQ analysis as shown in Fig 1A. To confirm this observation, we obtained primary Lig cells and OS-Lig cells from TOLF tissues, and examined PTGR1 expressions by western blot and RT-PCR analysis. We observed that the mRNA expressions of PTGR1 in OS-Lig cells increased by 2.3 fold compared with that in Lig cells (P<0.05) as shown in Fig 1B. Western blot results showed that protein expression of PTGR1 was significantly higher in OS-Lig cells than Lig cells (P<0.05) as shown in Fig 1C. These findings were consistent with our iTRAQ result of PTGR1.

### Effect of PTGR1 on cell proliferation

**Fig 2:**
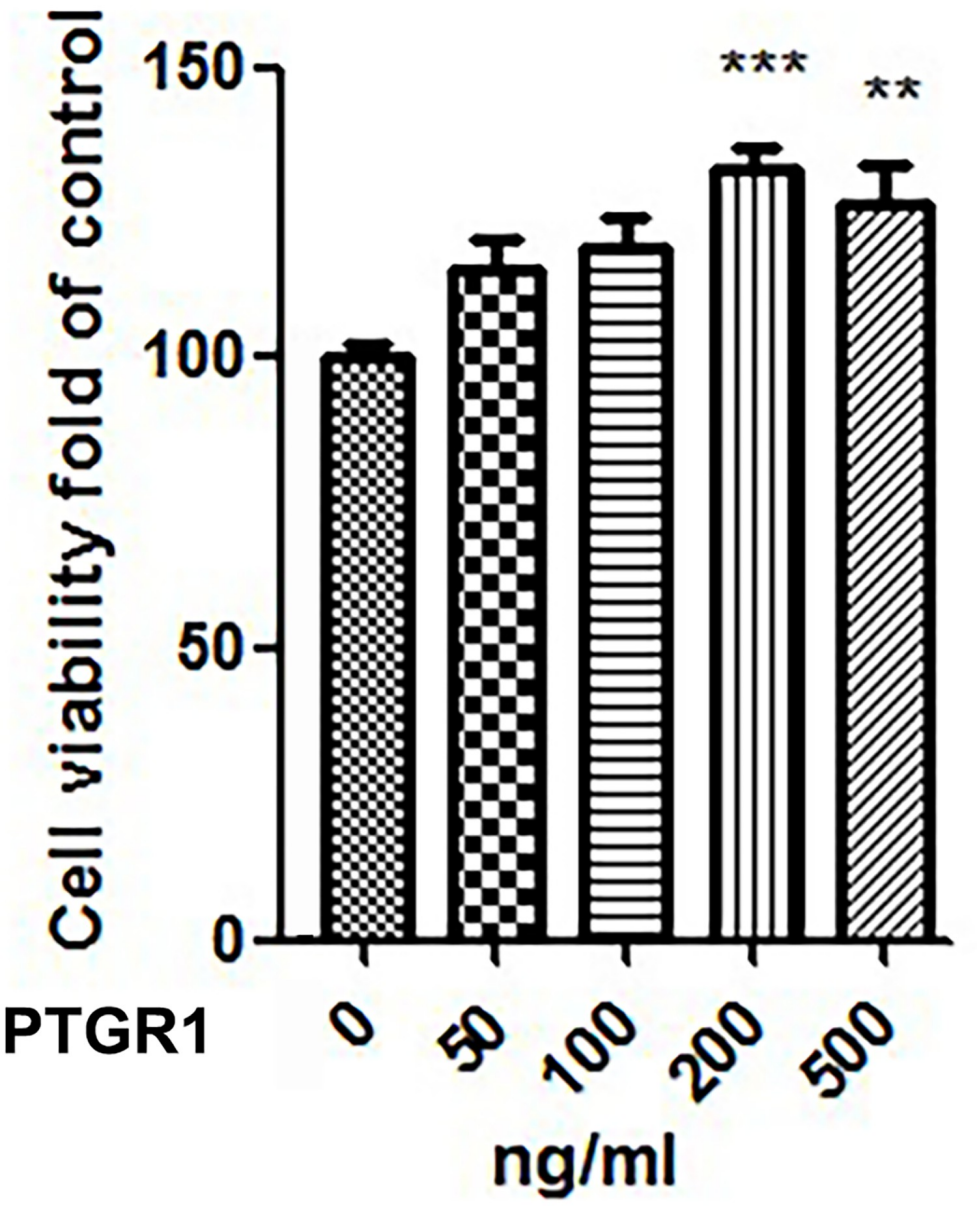
PTGR1 promotes cell proliferation in TOLF primary cells. Primary cells of TOLF were treated with various concentrations of PTGR1 for 24 h in a dose-dependent manner. Cell proliferation was determined by CCK8 assays.

The role of PTGR1 in TOLF remains unclear. Our previous study indicated that primary cells grew faster in the ossified ligamentum flavum of TOLF compared with the control [10]. It was reported that knockdown of PTGR1 inhibited cell proliferation of multiple cancer cell lines [16]. We hypothesize that PTGR1 may participate in cell proliferation in TOLF. To test this hypothesis, TOLF primary cells were treated for 24h with medium alone and medium containing PTGR1 at different concentrations. Cell proliferation was assessed using CCK8 cell proliferation assays. We found that the addition of PTGR1 led to increased cell proliferation in a dose-dependent manner as shown in Fig 2. We observed that the concentration of 200ng/mL of PTGR1 had the most obvious effect on cell proliferation compared with other concentrations.

### PTGR1 activated the expression of c-Myc

**Fig 3:**
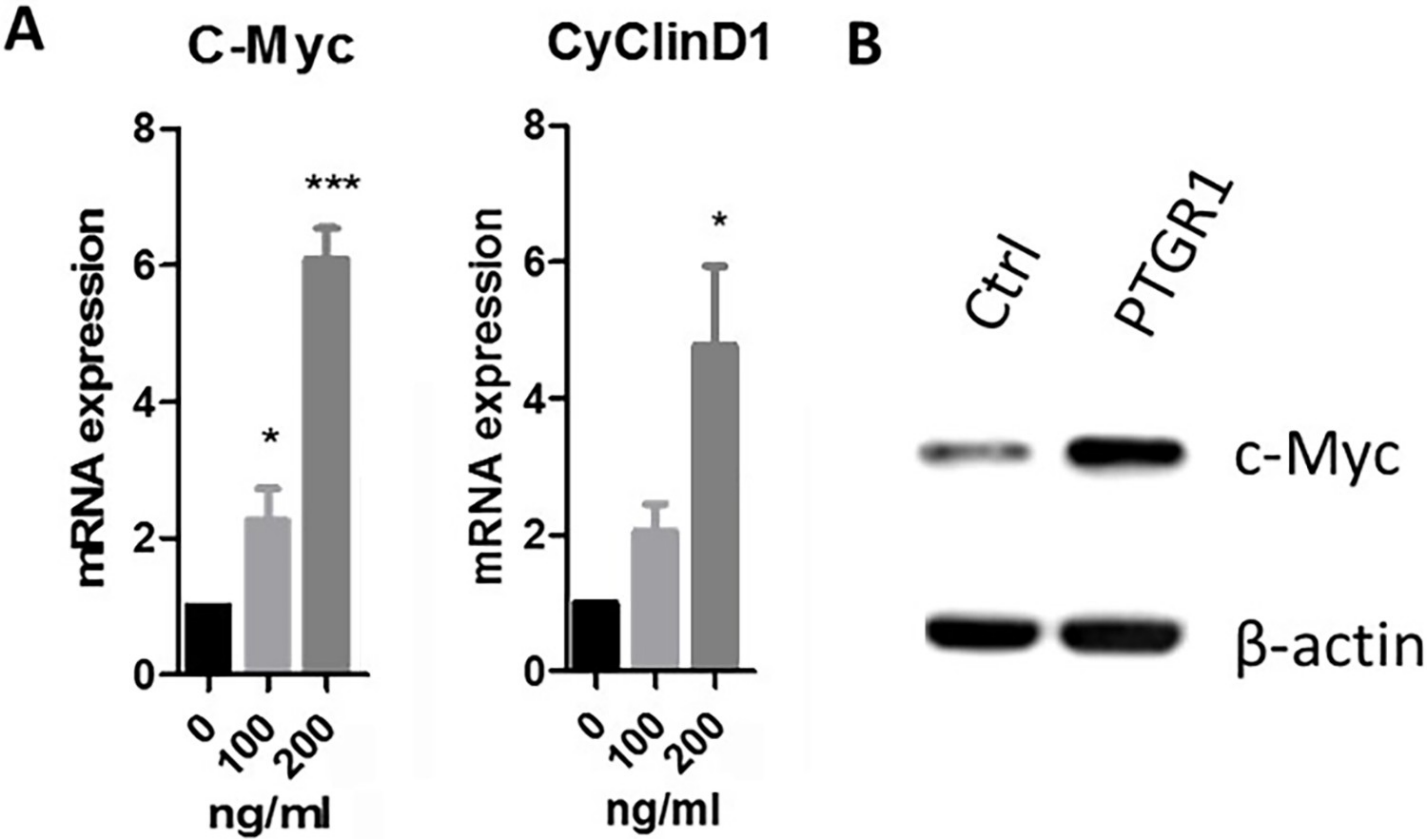
PTGR1 activates the expressions of cell proliferation related genes. Primary cells of TOLF were cultured and treated with PTGR1 for 24 h. Total RNA was isolated and measured by real time RT-PCR. The RNA level from the control group was normalized to a value of 1. Values were presented as the mean ±S.D. A paired t-test was performed comparing control and TNF-α group. A) Effect of PTGR1 on c-Myc expression and Cyclin D1 expression; B) Western blot analysis of c-Myc in primary cells after PTGR1 (200 ng/ml) treatment for 24 h.

Next, we examined the effect of PTGR1 on cell proliferation related genes in TOLF primary cells. Primary cells were treated with medium alone or medium containing 100, 200 ng/ml PTGR1 for 24h. Total RNA were isolated and measured by real time RT-PCR. As shown in Fig 3A, PTGR1-treatment enhanced mRNA expression of c-Myc by 6.1 fold at the concentration of 200ng/ml. Cyclin D1 expression increased by 4.8 fold in the presence of 200ng/ml PTGR1. At the protein level, western blot analysis revealed that PTGR1-treated groups had higher expression levels of c-Myc than the untreated group as shown in Fig 3B.

### PTGR1 activated the expression of c-Myc through JNK pathway

**Fig 4:**
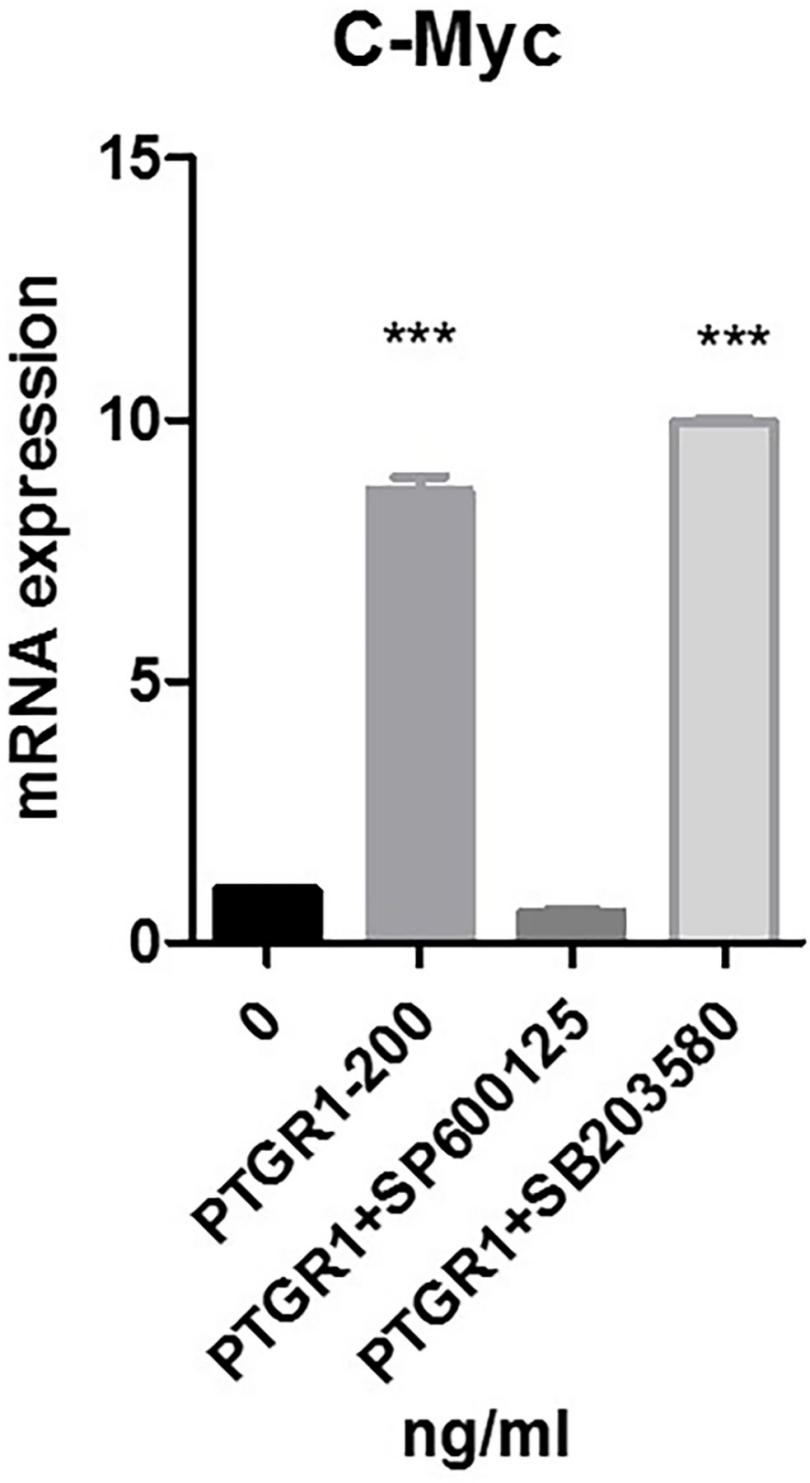
PTGR1 activated the expression of c-Myc through JNK pathway. Primary cells of TOLF were treated with 200ng/ml of PTGR1 for 24h. SP600125 and SB203580 were added to the culture medium 2h before PTGR1 treatment. Total RNA was isolated and expression of c-Myc was examined by qPCR.

Moreover, we explored which pathway was involved in c-Myc gene expression induced by PTGR1. By examining possible pathways involved in PTGR1 effect on c-Myc expression in TOLF primary cells, loss-of-function analysis was used to explore molecular mechanisms. The following inhibitors were used: SB203580 is a selective p38 MAPK inhibitor, and SP600125 is a selective JNK inhibitor. TOLF primary cells were treated with 200 ng/ml of PTGR1. Different inhibitors were used in the culture medium as indicated. As shown in Fig 4, PTGR1 treatment activated c-Myc expression by 8.6-fold. Addition of SP600125 almost abolished the activation of c-Myc expression induced by PTGR1. C-Myc activation by PTGR1 remained unchanged after addition of SB203580. These data suggest that PTGR1 activated the expression of c-Myc through JNK pathway.

## Discussion

TOLF is characterized by pathological heterotopic ossification in the ligamentum flavum. Recent studies have started to consider that TOLF progression was influenced by inflammatory factors. By iTRAQ analysis, we have already found ten inflammatory factors related to TOLF, including TNF-α and PTGR1 [17]. Our previous studies have demonstrated that TNF-α is involved in ossified ligamentum flavum in TOLF, and inflammatory factor stimulation resulted in the increased expressions of osteoblastic genes, such as BMP2, OCN, and ALP [10, 17].

Bone formation involves mesenchymal stem cell differentiation into osteoblast lineage [21]. Osteoblast differentiation is regulated by several transcription factors such as Ihh, Runx2, and Osx [22]. Both cell proliferation and cell differentiation are important for the process of bone formation. In our previous study, we demonstrated that primary cells from the ossified ligamentum flavum of TOLF (OS-Lig cells) grew faster than Lig cells, and that TNF-α regulated cell proliferation via cyclin D1 and c-Myc [10]. The expressions of cell proliferation factors are also enhanced in ligamentum flavum cells when they are stimulated with IL-6 [11]. These findings may partially explain the clinical observation of ligamentum flavum proliferous hypertrophy and hyperplasia in TOLF patients. However, we cannot rule out other possible factors involved in cell proliferation regulation in TOLF. Interestingly, some research groups have indicated that PTGR1 could contribute to proliferation in liver tumor cells. However, it remains unclear whether PTGR1 can affect pathological heterotopic ossification in ligamentum flavum and its potential mechanism. This study addressed the underlying mechanism of PTGR1 upregulation in TOLF.

To verify the iTRAQ result of PTGR1, we compared the expression level of PTGR1 in OS-Lig cells with Lig cells in this study. Indeed, the expression level of PTGR1 increased in OS-Lig cells. Both the RNA level and protein level exhibited the same outcome of increase as shown in Fig 1. Hence, it is predictable that PTGR1 may play a role in TOLF. The mechanism by which PTGR1 acts in TOLF has not been investigated yet. Previous studies indicated that PTGR1 was present in several cancer progresses. It was showed that PTGR1 reduced the time required for cell proliferation liver carcinogenesis [15]. Another group’s research showed that knockdown of PTGR1 inhibited cell proliferation of multiple cancer cell line [16].

c-Myc is a key target gene that plays an important role in various types of cell pathways [10]. Studies have shown that Cyclin D1 promotes cell proliferation. According to these results, we speculated that PTGR1 may effect TOLF via cell proliferation. In this study, a CCK8 assay was performed to support our speculation that PTGR1 promoted cell proliferation of primary cells of TOLF (Fig 2). Our observations showed that PTGR1 activated the mRNA expression of c-Myc and CyclinD1 (Fig 3A), and that the protein level of c-Myc increased on the concentration of 200 ng/ml PTGR1 (Fig 3B). Furthermore, blocking JNK pathway by selective JNK inhibitor SP600125 eliminated the positive effect of PTGR1 on c-Myc expression, indicating that PTGR1 activated the expression of c-Myc via JNK pathway (Fig 4).

Taken together, our new findings suggest that PTGR1 is involved in cell proliferation of TOLF. As far as we know, this is the first study to reveal the involvement of PTGR1 in thoracic ossification of the ligamentum flavum.

## Supporting information

S1 Raw images (PDF)

## Abbreviations

TOLF: Thoracic ossification of the ligamentum flavum
PTGR1: Prostaglandin reductase 1
iTRAQ: isobaric tags for relative and absolute quantitation
CCK8: Cell Counting Kit-8
GAPDH: glyceraldehyde-3-phosphate dehydrogenase

## References

[1] Sakamaki T, Sairyo K, Sakai T, Tamura T, Okada Y, Mikami H. Measurements of ligamentum flavum thickening at lumbar spine using MRI. Arch Orthop Trauma Surg. 2009;129(10):1415–9. doi: 10.1007/s00402-009-0849-1 19280205

[2] Fan D, Chen Z, Chen Y, Shang Y. Mechanistic roles of leptin in osteogenic stimulation in thoracic ligament flavum cells. The Journal of biological chemistry. 2007;282(41):29958–66. doi: 10.1074/jbc.M611779200 17702747

[3] Feng FB, Sun CG, Chen ZQ. Progress on clinical characteristics and identification of location of thoracic ossification of the ligamentum flavum. Orthopaedic surgery. 2015;7(2):87–96. doi: 10.1111/os.12165 26033987 PMC6583125

[4] Ning S, Chen Z, Fan D, Sun C, Zhang C, Zeng Y, et al. Genetic differences in osteogenic differentiation potency in the thoracic ossification of the ligamentum flavum under cyclic mechanical stress. International journal of molecular medicine. 2017;39(1):135–43. doi: 10.3892/ijmm.2016.2803 28004120 PMC5179181

[5] Zhao W, Shen C, Cai R, Wu J, Zhuang Y, Cai Z, et al. Minimally invasive surgery for resection of ossification of the ligamentum flavum in the thoracic spine. Wideochir Inne Tech Maloinwazyjne. 2017;12(1):96–105. doi: 10.5114/wiitm.2017.66473 28446938 PMC5397543

[6] Lang N, Yuan HS, Wang HL, Liao J, Li M, Guo FX, et al. Epidemiological survey of ossification of the ligamentum flavum in thoracic spine: CT imaging observation of 993 cases. European spine journal: official publication of the European Spine Society, the European Spinal Deformity Society, and the European Section of the Cervical Spine Research Society. 2013;22(4):857–62. doi: 10.1007/s00586-012-2492-8 22983651 PMC3631053

[7] Yayama T, Uchida K, Kobayashi S, Kokubo Y, Sato R, Nakajima H, et al. Thoracic ossification of the human ligamentum flavum: histopathological and immunohistochemical findings around the ossified lesion. Journal of neurosurgery Spine. 2007;7(2):184–93. doi: 10.3171/SPI-07/08/184 17688058

[8] Xue L, Zhu Z, Wang Z, Li H, Zhang P, Wang Z, et al. Knockdown of prostaglandin reductase 1 (PTGR1) suppresses prostate cancer cell proliferation by inducing cell cycle arrest and apoptosis. Biosci Trends. 2016;10(2):133–9. doi: 10.5582/bst.2016.01045 27150108

[9] Maigne JY, Ayral X, Guerin-Surville H. Frequency and size of ossifications in the caudal attachments of the ligamentum flavum of the thoracic spine. Role of rotatory strains in their development. An anatomic study of 121 spines. Surgical and radiologic anatomy: SRA. 1992;14(2):119–24. 1641735 10.1007/BF01794886

[10] Zhang C, Chen Z, Meng X, Li M, Zhang L, Huang A. The involvement and possible mechanism of pro-inflammatory tumor necrosis factor alpha (TNF-alpha) in thoracic ossification of the ligamentum flavum. PloS one. 2017;12(6):e0178986. 28575129 10.1371/journal.pone.0178986 PMC5456390

[11] Huang AY, Shu L, Chen Z, Zhang C. IL-6 is involved in thoracic ossification of the ligamentum flavum. PloS one. 2022;17(7):e0272357. doi: 10.1371/journal.pone.0272357 35905126 PMC9337630

[12] Li F, Chen Q, Xu K. Surgical treatment of 40 patients with thoracic ossification of the ligamentum flavum. Journal of neurosurgery Spine. 2006;4(3):191–7. doi: 10.3171/spi.2006.4.3.191 16572617

[13] Gao R, Yuan W, Yang L, Shi G, Jia L. Clinical features and surgical outcomes of patients with thoracic myelopathy caused by multilevel ossification of the ligamentum flavum. Spine J. 2013;13(9):1032–8. doi: 10.1016/j.spinee.2013.02.034 23541451

[14] Kawaguchi Y, Yasuda T, Seki S, Nakano M, Kanamori M, Sumi S, et al. Variables affecting postsurgical prognosis of thoracic myelopathy caused by ossification of the ligamentum flavum. Spine J. 2013;13(9):1095–107. doi: 10.1016/j.spinee.2013.03.001 23602378

[15] Sanchez-Rodriguez R, Torres-Mena JE, Quintanar-Jurado V, Chagoya-Hazas V, Rojas Del Castillo E, Del Pozo Yauner L, et al. Ptgr1 expression is regulated by NRF2 in rat hepatocarcinogenesis and promotes cell proliferation and resistance to oxidative stress. Free Radic Biol Med. 2017;102:87–99. doi: 10.1016/j.freeradbiomed.2016.11.027 27867096

[16] Wang X, Yin G, Zhang W, Song K, Zhang L, Guo Z. Prostaglandin Reductase 1 as a Potential Therapeutic Target for Cancer Therapy. Front Pharmacol. 2021;12:717730. doi: 10.3389/fphar.2021.717730 34421612 PMC8377670

[17] Wang B, Chen Z, Meng X, Li M, Yang X, Zhang C. iTRAQ quantitative proteomic study in patients with thoracic ossification of the ligamentum flavum. Biochemical and biophysical research communications. 2017;487(4):834–9. doi: 10.1016/j.bbrc.2017.04.136 28455229

[18] Yang X, Chen Z, Meng X, Sun C, Li M, Shu L, et al. Angiopoietin-2 promotes osteogenic differentiation of thoracic ligamentum flavum cells via modulating the Notch signaling pathway. PloS one. 2018;13(12):e0209300. doi: 10.1371/journal.pone.0209300 30557327 PMC6296551

[19] Zhang C, Cho K, Huang Y, Lyons JP, Zhou X, Sinha K, et al. Inhibition of Wnt signaling by the osteoblast-specific transcription factor Osterix. Proc Natl Acad Sci U S A. 2008;105(19):6936–41. doi: 10.1073/pnas.0710831105 18458345 PMC2383965

[20] Huang AY, Xiong Z, Liu K, Chang Y, Shu L, Gao G, et al. Identification of kaempferol as an OSX upregulator by network pharmacology-based analysis of qianggu Capsule for osteoporosis. Front Pharmacol. 2022;13:1011561. doi: 10.3389/fphar.2022.1011561 36210811 PMC9539404

[21] Zhang C. Transcriptional regulation of bone formation by the osteoblast-specific transcription factor Osx. Journal of orthopaedic surgery and research. 2010;5:37. doi: 10.1186/1749-799X-5-37 20550694 PMC2898801

[22] Zhang C. Molecular mechanisms of osteoblast-specific transcription factor Osterix effect on bone formation. Beijing Da Xue Xue Bao. 2012;44(5):659–65. 23073571

