# Supplementary figures and images for: PTGR1 is involved in cell proliferation in thoracic ossification of the ligamentum flavum
Source: PLoS One. 2023 Nov 1;18(11):e0292821. doi: 10.1371/journal.pone.0292821 (PMC10619815; doi:10.1371/journal.pone.0292821)

Fig 1C raw image

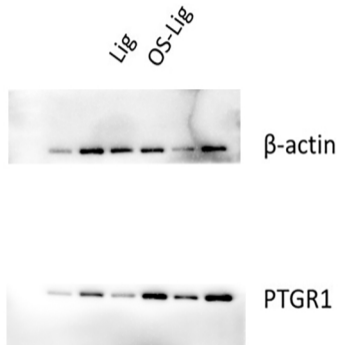

Fig 3B raw image

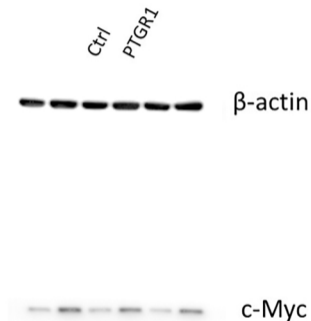

Supplement: S1 Raw images — (PDF) [file pone.0292821.s001.pdf]
